
JOURNAL INFORMATION
==============================
NLM Title Abbreviation: ScientificWorldJournal
Journal ID: ScientificWorldJournal
NLM Journal ID: 101131163
Journal ID: TSWJ

The Scientific World Journal

ISSN: 2356-6140
EISSN: 1537-744X
Publisher: Wiley

ARTICLE INFORMATION
==============================
PMCID: PMC6009750
PMID: 29973804
DOI: 10.1100/tsw.2002.30
Article ID: 734812
Article version: 1
Subjects: Short Report

Mechanism of Unfolding of A Model Helical Peptide

Pastrana-Rios Belinda *
Lopez Gustavo
Department of Chemistry, University of Puerto Rico Mayagüez Campus, Mayagüez, Puerto Rico 00681-9019, USA
*Belinda Pastrana-Rios: belinda@hpcf.upr.edu
Publication date: 2002
Electronic publication date: 2002 Jan 31
Volume: 2
First page: 59
Last page: 61
Copyright: Copyright © 2002 Belinda Pastrana-Rios and Gustavo Lopez.
Copyright year: 2002
License: This is an open access article distributed under the Creative Commons Attribution License, which permits unrestricted use, distribution, and reproduction in any medium, provided the original work is properly cited.
License URL: https://creativecommons.org/licenses/by/3.0/

Funding: National Institutes of Health

==============================
Advances in Gene Technology: The Genome and Beyond –
Structural Biology for Medicine (Proceedings of the 2002 Miami
*Nature Biotechnology* Winter Symposium)
*TheScientificWorld* 2002, 2(S2), 59–61
ISSN 1532-2246; DOI 10.1100/tsw.2002.30

### MECHANISM OF UNFOLDING OF A MODEL HELICAL PEPTIDE

Belinda Pastrana-Rios and Gustavo López
Department of Chemistry, University of Puerto Rico Mayagüez Campus, Mayagüez, Puerto Rico
00681-9019
Belinda@hpcf.upr.edu

**INTRODUCTION.** Synthetic model helical peptides, Acetyl-W(EAAAR)$_5$A-amide with $^{13}$C=O specifically labeled alanine segments in repeats n = 1,2 or 4,5 were studied in aqueous $D_2O$ solution as a function of temperature using Fourier transform infrared spectroscopy and two-dimensional correlation analysis. The $^{13}$C=O provided a probe which was sensitive to the carbonyl stretch in the peptide bonds of the alanine residues at the amino terminal end in one peptide as compared to the probe in the carboxy terminal end of the other peptide during thermal perturbation. The relative stability of each terminal end was examined; the more stable terminal was determined to be the amino terminal end. The 27-residue peptide was also simulated using parallel-tempering Monte Carlo simulations in the canonical ensemble. The peptide was modeled using the spring-bead interparticle potential coupled to a modified Lennard-Jones (L-J) potential that described the nonbonded interactions. The hydrogen bonding and salt bridge interactions were modeled by adjusting the attractive and repulsive parts of the L-J potential. Conformational changes were characterized by increments in the constant volume heat capacity, internal energy, and the radius of gyration of the peptide.

**METHOD.** FT-IR experiments involved the use of fully H$\rightarrow$D exchanged peptide redissolved in 1 mM phosphate buffer, 10 mM NaCl in 99.9% D, $D_2O$ at pD = 6.6. $CaF_2$ cells and a dual-chamber, custom-milled cell holder connected to a Neslab circulating bath for temperature variation were used with an FTS-40 Bio-Rad (Cambridge, MA) equipped with custom shuttle and interface. Typically, 512 scans were coadded, apodized with a triangular function, and Fourier transformed to provide a resolution of 4 cm$^{-1}$, with data encoded every 2 cm$^{-1}$. Sixteen spectra were collected at sequential increments of temperature, allowing for thermal equilibrium. The spectra (in the spectral region of interest 1520–1750 cm$^{-1}$ and 1300–1400 cm$^{-1}$) were then analyzed using two-dimensional FT-IR correlation and curve-fitting.

Two-dimensional FT-IR correlation analysis was performed using MathCad 2000 Professional (MathSoft, Inc., Cambridge, MA) software. The curve-fitting routines were done using Grams 3.01 (Galactic Industries Corp., Salem, NH) program. Origin 6 program was used to plot curve-fitted data.

Simulations were carried out using an SGI Origin 200.

**RESULTS.** Structural and dynamic changes were investigated using FT-IR Isotope edited spectroscopy, two-dimensional correlation analysis (see Fig. 1) and molecular simulations. The arginine-glutamate salt bridge provides additional stability to the helical structure of this model peptide rendering it as a solid cylinder.

The molecular simulations results agree very well with the experimental data obtained in that two conformational changes were observed. The first one occurs at low temperatures and corresponds to the unwinding of the amino terminal end and the second transition is associated to the unwinding of the carboxyl terminal end. A third transition is observed in the simulation at high temperature, which is associated to the random coil structure of the peptide.

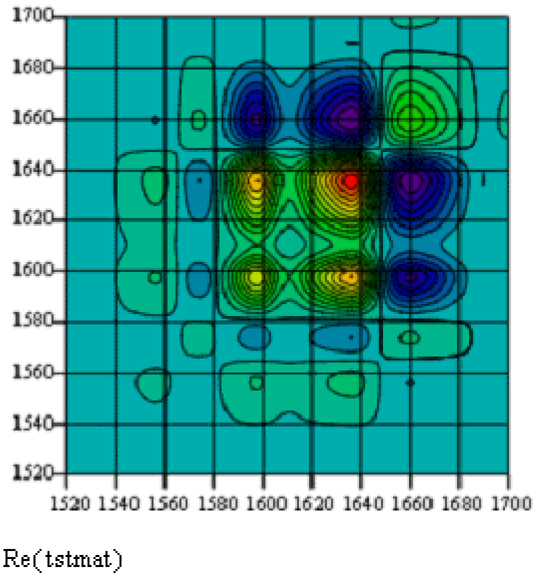

Wavenumber $\nu_1$ vs Wavenumber $\nu_2$¶

**FIGURE 1.** Two-dimensional correlation Synchronous plot for the n = 4,5 $^{13}$C=O labeled peptide below the transition temperature (T = 0–40$^{o}$C).

**DISCUSSION.** The α-helix as a structural motif is very common among proteins, by studying this model helical peptide one can gain insight in the stability of the α-helix and thus carry this information over to proteins with secondary structure motifs comprised of helices. The carboxy terminal end of the helix is less stable than the amino terminal end due to the additive effect of the carbonyl dipoles in the structure. Furthermore, The phase information obtained suggests that the salt bridge between the glutamates (1567 cm$^{-1}$) and the arginines (1606 and 1596 cm$^{-1}$) break prior to the helix unwinding (1599 cm$^{-1}$, $^{13}$C labeled α-helix band) to generate the denatured peptide. In addition, the glutamate cross peak was correlated to the auto peaks of the unlabeled α-helix and random coil, suggesting a strong interaction and thus defining the cooperativity during thermal denaturation process, i.e., one event must occur prior to the other in the order described above with a strong interaction between these vibrational modes.

The use of FT-IR spectroscopy, two-dimensional correlation analysis and molecular simulations has proven useful towards the understanding of dynamic processes that occur at the structural level.

**ACKNOWLEDGMENT.** The author sincerely thanks Dr. Frank Prendergast for the generous supply of the $^{13}C$ labeled peptide. The author would also thank Dr McCormick's group in the synthesis and TOF-MS for the specifically labeled Ac-W(EAAAR)$_5$A-NH$_2$. The work was supported in part by NIH-MBRS-SCORE

### REFERENCES

1. Merutka, G., Dimitrios, M., Bruschweiler, R., and Stellwagen, E. (1993) Biochemistry 32, 13089–13097.
2. Shalongo, W. and Stellwagen, E. (1995) Protein Sci. 4, 1161–1166.
3. Shalongo, W., Dugad, L., and Stellwagen, E. (1994) J. Am. Chem. Soc. 116, 2500–2508.
4. Stellwagen, E. and Shalongo, W. (1998) Biopolymers 43, 413–418.
5. Noda, I. (1989) J. Am. Chem. Soc. 111, 8116–8118.
6. Graff, D.K., Pastrana-Rios, B., Venyaminov, S.Y., and Prendergast, F.G. (1997) J. Am. Chem. Soc. 119, 11282–11294.
7. Pastrana-Rios, B. (2001) Biochemistry 40, 9074–9081.
